# 3D-SIM Super Resolution Microscopy Reveals a Bead-Like Arrangement for FtsZ and the Division Machinery: Implications for Triggering Cytokinesis

**DOI:** 10.1371/journal.pbio.1001389

**Published:** 2012-09-11

**Authors:** Michael P. Strauss, Andrew T. F. Liew, Lynne Turnbull, Cynthia B. Whitchurch, Leigh G. Monahan, Elizabeth J. Harry

**Affiliations:** The ithree institute, University of Technology Sydney, Sydney, New South Wales, Australia; MRC Laboratory of Molecular Biology, United Kingdom

## Abstract

FtsZ is a tubulin-like GTPase that is the major cytoskeletal protein in bacterial cell division. It polymerizes into a ring, called the Z ring, at the division site and acts as a scaffold to recruit other division proteins to this site as well as providing a contractile force for cytokinesis. To understand how FtsZ performs these functions, the in vivo architecture of the Z ring needs to be established, as well as how this structure constricts to enable cytokinesis. Conventional wide-field fluorescence microscopy depicts the Z ring as a continuous structure of uniform density. Here we use a form of super resolution microscopy, known as 3D-structured illumination microscopy (3D-SIM), to examine the architecture of the Z ring in cells of two Gram-positive organisms that have different cell shapes: the rod-shaped *Bacillus subtilis* and the coccoid *Staphylococcus aureus*. We show that in both organisms the Z ring is composed of a heterogeneous distribution of FtsZ. In addition, gaps of fluorescence were evident, which suggest that it is a discontinuous structure. Time-lapse studies using an advanced form of fast live 3D-SIM (Blaze) support a model of FtsZ localization within the Z ring that is dynamic and remains distributed in a heterogeneous manner. However, FtsZ dynamics alone do not trigger the constriction of the Z ring to allow cytokinesis. Lastly, we visualize other components of the divisome and show that they also adopt a bead-like localization pattern at the future division site. Our data lead us to propose that FtsZ guides the divisome to adopt a similar localization pattern to ensure Z ring constriction only proceeds following the assembly of a mature divisome.

## Abstract 2

Super resolution three-dimensional imaging reveals a new picture of how bacterial cell division proteins localize to the division site, including the formation of dynamic bead-like patterns, and explains how the division ring constricts.

## Author Summary

Because bacterial cells are so small, it is challenging to image the spatial organization of proteins inside them. All the proteins that orchestrate cell division in these organisms localize to the division site prior to division, but it has not so far been possible to obtain a clear highresolution three-dimensional picture of the dynamics of their localization. In this study we use a new type of super resolution microscopy called three-dimensional structured illumination microscopy (3D-SIM) to analyze the localization of proteins involved in cell division in two types of bacteria that have different cell shapes: the rod-shaped *Bacillus subtilis* and the spherical *Staphylococcus aureus*. We show that FtsZ, a cytoskeletal protein that serves as a scaffold for the cytokinetic ring, localizes to the division site in a dynamic bead-like pattern, rather than a uniform ring as was previously proposed, in both types of bacteria. Our observations also provide an explanation of how this ring constricts to split a bacterial cell in two and suggests that this spatial organization of division proteins is conserved among bacteria and is crucial for the regulation of this central cellular process.

## Introduction

Cell division is essential for the propagation of all living species. Division in many prokaryotic organisms relies on the polymerization of the protein FtsZ into a ring structure, called the Z ring, at the division site. FtsZ is found in virtually all bacterial species, many species of archaea, and even in higher plants where FtsZ is involved in chloroplast division [1],[2]. In bacteria, the ability of FtsZ to assemble into the Z ring marks the beginning of the division process [3]. Following its formation on the inner cell membrane the Z ring acts as a scaffold to recruit the other cell division proteins to this site [4],[5]. Several studies have indicated that the Z ring has an additional role in providing the contractile force required to “pull in” the cell envelope during cytokinesis [6],[7],[8],[9].

FtsZ is homologous to eukaryotic tubulin and they belong to a distinct family of GTPases which share similar tertiary structure [3],[10],[11]. FtsZ subunits can assemble into single-stranded protofilaments in a GTP-dependent manner [12],[13]. These FtsZ protofilaments can also form higher order structures such as ribbons, tubules, and other types of polymers in vitro [14],[15],[16],[17],[18]. The formation of these different higher order structures has been suggested to play a role in the formation of the Z ring and its ability to constrict during cytokinesis. However, the various FtsZ structures form under various experimental conditions that do not often mimic conditions in vivo. Thus knowing how FtsZ assembles into physiologically relevant structures inside the bacterial cell is vital for establishing how the Z ring forms and subsequently how it constricts in vivo.

Conventional fluorescence microscopy, using either immunofluorescence in fixed cells or FtsZ-GFP fusion proteins in live cells, shows the Z ring as a uniformly labeled fluorescent band, suggesting a single continuous structure of uniform density. While conventional electron microscopy has failed to detect the Z ring in bacterial cells, more recent electron cryotomography (ECT) studies have shown the Z ring of the Gram-negative marine bacterium, *Caulobacter crescentus*, to be a largely disconnected structure composed of sparse and irregularly distributed filaments of FtsZ [19]. This is in stark contrast to the continuous, uniform Z ring structure observed by conventional fluorescence microscopy. Unfortunately, ECT studies cannot be performed in other well-studied model bacteria such as *Bacillus subtilis* and *Escherichia coli* because the cells are either too thick or the Z ring cannot be detected [20]. An outstanding question that remains is whether the Z ring is a continuous or discontinuous structure [21]. Determining the structure of the Z ring has implications towards understanding how the structure of the Z ring changes during constriction.

Although conventional fluorescence microscopy has not been able to answer this question, it is still the most widely used method to examine FtsZ structures because it offers several advantages over electron microscopy. Most notable is the ability to specifically label the protein of interest and to visualize proteins in live, untreated cells using GFP fusions. These fusion proteins have demonstrated that the Z ring is actually assembled from helical FtsZ precursor structures in *B. subtilis*, *E. coli* and *C. crescentus* [22],[23],[24],[25]. Furthermore, the use of fusion proteins in a technique known as fluorescence recovery after photo-bleaching (FRAP) has shown that the Z ring is in fact a highly dynamic structure, constantly undergoing subunit turnover throughout its lifetime [26],[27]. Taken together it is clear how valuable it is to visualize a specifically labeled protein of interest in live cells. However, conventional fluorescence microscopy is limited by the diffraction barrier, which affects our ability to clearly visualize how FtsZ assembles into the Z ring and how it is organized within the Z ring.

New forms of high resolution fluorescence-based imaging have recently been developed and are collectively known as super resolution microscopy [28]. These techniques break the diffraction barrier through various means of point-spread-function engineering. The end result allows visualization of sub-cellular structures at much higher resolution, while still maintaining all the advantages of conventional wide-field fluorescence microscopy. Recently, super resolution microscopy techniques such as photoactivated localization microscopy (PALM) and stimulated emission depletion (STED) microscopy have been used to examine FtsZ localization in *E. coli* and *B. subtilis*, respectively [29],[30],[31]. Despite the substantial increase in sub-diffraction resolution provided by these imaging modalities, PALM and STED do not depict the Z ring as a discontinuous structure in the cell [29],[30],[31]. One of the major limitations in these techniques is that they only improve the lateral image resolution obtained from the sample and consequently they could not determine the whole 3D architecture of the Z ring [29],[30],[31].

Another super resolution fluorescence microscopy technique, known as 3D-structured illumination microscopy, or 3D-SIM, allows complete 3D visualization of structures inside cells. 3D-SIM is the only form of super resolution microscopy that offers a 2-fold increase in both lateral and axial resolution to generate true 3D super resolution images [32]. Here we report the use of 3D-SIM to visualize the Z ring in two Gram-positive organisms: rod-shaped *B. subtilis* and spherical *Staphylococcus aureus*. We show that the overall structure of the Z ring in both organisms is very similar and composed of a heterogeneous distribution of FtsZ, suggesting a discontinuous Z ring structure. The increased axial resolution capabilities of 3D-SIM uniquely allow visualization of the localization of FtsZ within the Z ring as well as the dynamic changes that occur to this localization in live cells over time using a new form of fast live 3D-SIM, known as OMX Blaze. The dynamic changes in FtsZ localization within the Z ring support the previously proposed iterative pinching model for constriction, but they are not responsible for triggering this process. Finally we visualize the localization of other divisome proteins to show that they share a similar heterogeneous and dynamic distribution at the site of cytokinesis.

## Results

### FtsZ Concentration within the Z Ring Is Heterogeneous

Our initial approach to visualize the 3D structure of the Z ring was performed in live cells of *B. subtilis* in a strain designated SU570. In this strain, the wild-type *ftsZ* gene has been replaced with an *ftsZ-gfp* fusion gene under the control of the native promoter (see Table 1) [33]. Consequently, the FtsZ-GFP fusion protein is the only source of FtsZ inside the cell capable of forming a Z ring. This enables direct visualization of all the FtsZ present in the cell and thus a genuine 3D image of the Z ring. At 30°C, we can confirm that *B. subtilis* SU570 is able to utilize the fusion protein as the sole source of FtsZ required for division [33].

**Table 1:** *S. aureus* and *B. subtilis* strains.

Strain	Description^a^	Source/Reference
*S. aureus* strains		
SH1000	NCTC-8325 derivative strain with restored sigma B activity	[63]
SA89	RN4220 strain carrying plasmid pLOWErmFtsZ-GFP and pGL485; Erm^r^, Cm^r^	[39]
SA98	SH1000 strain carrying plasmid pLOWErmFtsZ-GFP and pGL485; Erm^r^, Cm^r^	[39]
JGL227	SH1000 ezrA-GFP; Erm^r^	[48]
ELC2	SH1000 derivative with the protein A locus replaced with a Tetracycline resistance marker; Tet^r^	[64]
SA126	JGL227 strain transduced into the ELC2 background carrying plasmid pGL485; Erm^r^, Cm^r^, Tet^r^	This study
RNpPBP2-31	RN4220 strain GFP-PBP2	[51]
*B. subtilis* strains		
SU5	168 *trpC2*	E. Nester
PL91	PY79 *div-355*	[45]
SU347	168 *trpC2 div-355*	Lab stock^b^
SU568	168 *trpC2 amyE::Pspac-ftsZ-gfp* Cm^r^	Lab stock
SU570	168 *trpC2 ftsZ::ftsZ-gfp* Spec^r^	Lab stock
SU744	168 *trpC2 amyE::Pspac-ftsZ-gfp* Cm^r^ *div-355*	This study

a Antibiotic resistance markers present in the strain: Tet^r^, tetracycline resistance; Erm^r^, erythromycin resistance; Cm^r^, chloramphenicol resistance; Spec^r^, spectinomycin resistance.

b The *div-355* mutation was introduced into the SU5 background by congression with *glnA^+^*.

**Figure 1:**
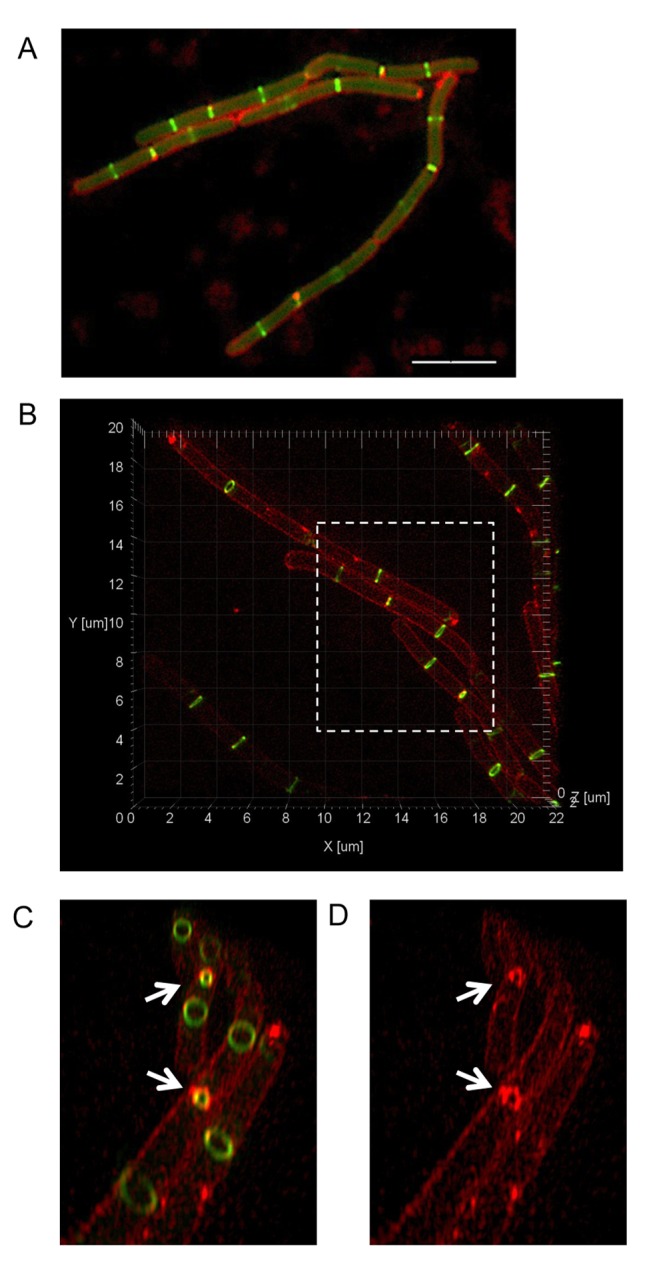
3D-SIM images of FtsZ-GFP localization in live cells of *B. subtilis*. (A) Conventional wide-field fluorescence microscopy (Zeiss) image of *B. subtilis* strain SU570 (*ftsZ-gfp*) stained with the membrane dye FM4-64 shows how FtsZ-GFP assembles into Z rings. Scale bar, 5 µm. (B) When the same strain is imaged using 3D-SIM (OMX V3), regions of interest from the image can be selected (dashed box) to zoom in and rotate the image around the *z*-axis to view 3D FtsZ structures in the axial plane. (C–D) The improved image resolution provided by 3D-SIM allows visualization of constricting Z rings and the inner the cell membrane during division (indicated by white arrows).

Figure 1A shows a typical image of FtsZ localization in SU570 using conventional wide-field fluorescence microscopy (Zeiss). The most conspicuous FtsZ structure is the Z ring that appears as a uniform transverse band when it is imaged in a single focal plane. A total of 240 individual images were acquired using 3D-SIM and subsequently reconstructed to generate a complete 3D fluorescence image. Figure 1B shows an example of a 3D-SIM image of SU570 cells that have also been stained with FM4-64 dye to visualize the membrane. The increase in both lateral (*x*-*y* axis) and axial (*z*-axis) resolution of 3D-SIM enabled visualization of very clear membrane stain and 3D ring structures of FtsZ that can be rotated and viewed from any angle (Figure 1C–D).

**Figure 2:**
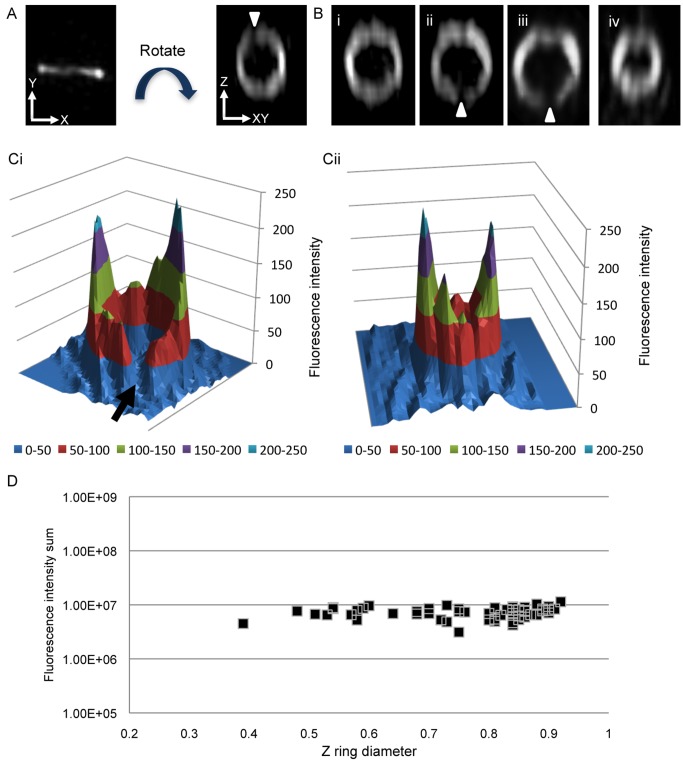
Examining the distribution of FtsZ inside the Z ring of *B. subtilis* in live SU570 (*ftsZ-gfp*) cells. (A) When the Z ring is visualized in live *B. subtilis* rod-shaped (SU570) cells, it appears as a transverse band of fluorescence. However, 3D-SIM (OMX V3) allows images of the Z ring to be rotated around the *z*-axis to clearly see how FtsZ is distributed within the Z ring in live cells of this strain. Small regions of low fluorescence intensity (gaps) in the Z ring are indicated by white arrowheads and cannot be seen without rotating the image. (Bi–iii) Additional examples of heterogeneous Z rings with a typical Z ring diameter of ∼0.9 µm. (Biv) A constricting Z ring with a diameter of 0.65 µm. (Ci) A typical 3D intensity profile reveals differences in fluorescence intensity and thus concentration of FtsZ-GFP in the Z ring with a diameter of 0.85 µm. The amount of fluorescence emanating from gaps is minimal and almost approaches baseline levels of fluorescence (black arrow). (Cii) A similar 3D intensity profile shows FtsZ-GFP distribution remains heterogeneous in a constricting Z ring; diameter, 0.65 µm. (D) The total fluorescence intensity of 56 different Z rings was analyzed. The relative amount of FtsZ-GFP fluorescence stays constant even as the diameter of the Z ring decreases.

The Z ring is normally visualized as a transverse band due to its orientation within *B. subtilis* cells (Figure 2Ai), but when the 3D-SIM image is rotated around the *z*-axis so that it is viewed in the axial plane, it is clear that the fluorescence intensity of the Z ring is not uniform throughout (Figure 2Aii and Movie S1). Rather, FtsZ-GFP appears heterogeneously distributed in all Z rings with a diameter between ∼300 and 900 nm (*n* = 84) (Figure 2Bi–iv). In the few constricting Z rings with a diameter less than ∼300 nm (*n* = 3), the Z ring appears as an intense focus in the center of the cell and it is difficult to clearly visualize the heterogeneous distribution of FtsZ-GFP in Z rings with such small physical dimensions (Figure S1). To confirm this image of the Z ring using 3D-SIM in cells containing only native FtsZ, we examined Z rings in wild-type *B. subtilis* cells using immunofluorescence (Figure S2). As with FtsZ-GFP, the distribution of native FtsZ in Z rings in wild type cells (*n* = 73) was heterogeneous and closely resembled the appearance of the Z ring when visualized using the FtsZ-GFP fusion.

Interestingly, we could visualize small regions of the Z ring where the fluorescence intensity of FtsZ-GFP (or FtsZ with immunofluorescence) is very low (indicated by white arrowheads in Figure 2A and 2B). These regions of low fluorescence intensity in the Z ring, which we will refer to as gaps, were observed in approximately 15% of all Z rings (*n* = 84). The gaps are approximately 118–200 nm in size and thus would not be resolved by conventional fluorescence microscopy. Moreover, visualization of these gaps requires that the 3D-SIM image is rotated around the *z*-axis and viewed in the axial plane in *B. subtilis*. To quantify the distribution of FtsZ-GFP within the Z ring we analyzed the image data using Figure 2Biii and 2Biv as examples (20 were analyzed in detail). The image data from these figures was used to generate 3D fluorescence intensity plots shown in Figure 2Ci and 2Cii (additional examples are presented in Figure S3). The intensity profiles of Z rings with gaps demonstrated that fluorescence could still be detected in these gaps (indicated by black arrow in Figure 2Ci). The vast majority of these gaps (93%) were observed in Z rings with a diameter of ∼800–900 nm. This correlation between the appearance of gaps in the Z ring and Z ring diameter prompted us to determine if the amount of FtsZ within the Z ring changes during constriction to fill in the gaps. The sum of FtsZ-GFP fluorescence intensity from 56 different Z rings was measured and plotted against ring diameter (Figure 2D). It is clear from this analysis that the total intensity of FtsZ in the Z ring remains relatively constant even when the diameter of the Z ring is decreasing. Thus the density of FtsZ in the ring becomes greater as the physical dimensions of the Z ring become smaller. However, due to resolution limits we cannot rule out the possibility that gaps continue to be generated in Z rings with a diameter less than 800 nm.

### 3D-SIM of Z Rings in *S. aureus* Cells Also Reveals a Heterogeneous Distribution of FtsZ

**Figure 3:**
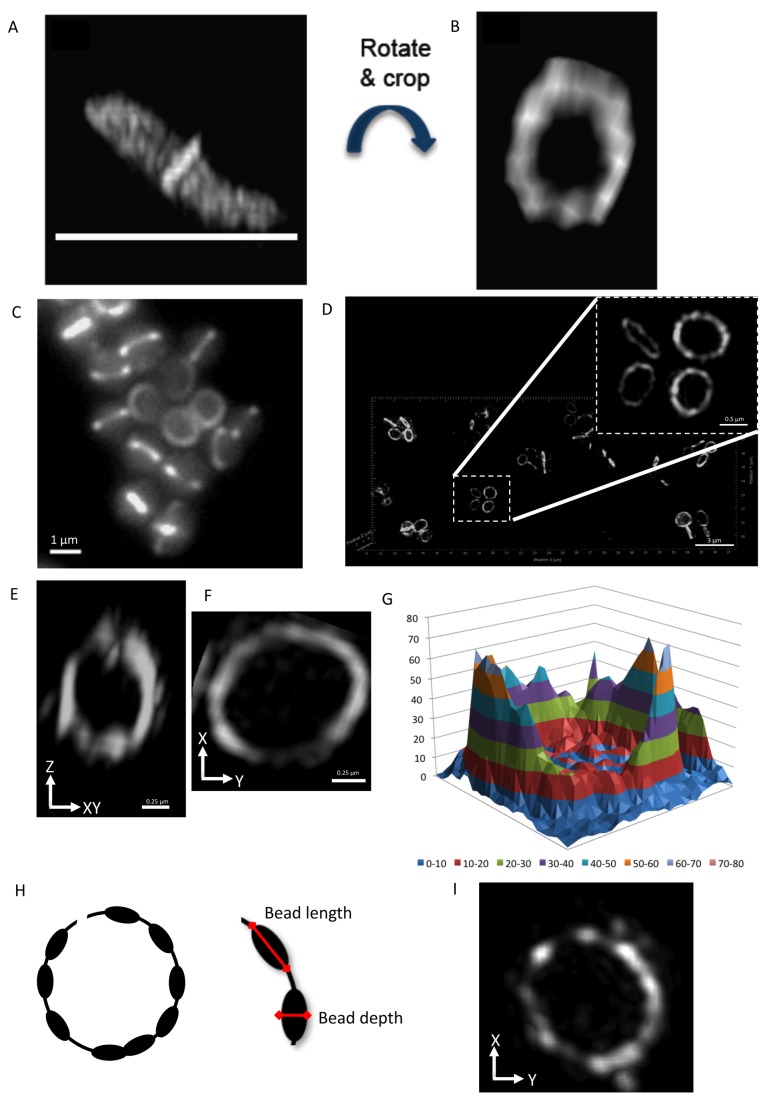
Z ring structure in *S. aureus* and *B. subtilis*. (A and B) When the Z ring is examined closer to the *x-y* orientation in *B. subtilis*, it reveals that it is heterogeneous throughout the entire Z ring (white line represents microscope slide). Z ring diameter, 0.8 µm. (C and D) *S. aureus* expressing FtsZ-GFP cells (SA94) visualized using conventional wide-field fluorescence image (Zeiss) and 3D-SIM (acquired using OMX V3), respectively. (E) The appearance of the Z ring in SA94 cells imaged in the axial orientation with 3D-SIM. (F) A close up image of a Z ring in SA94 cells imaged in the lateral orientation with 3D-SIM (OMX V3). (G) A 3D intensity plot shows how the relative abundance of FtsZ-GFP in all regions of the Z ring shown in panels (F) and (H). A graphical representation of the heterogeneous Z ring in *S. aureus*. Red arrows indicate dimensions used for measuring bead size and width in *S. aureus* cells. (I) 3D-SIM (OMX V3) image of the Z ring visualized using 1∶100 dilution of anti-FtsZ antibodies. *S. aureus* SA94 cells were grown in L-broth induced with 0.05 mM IPTG.

It is important to note that the heterogeneous FtsZ staining and gaps seen within the *B. subtilis* Z ring were more apparent in some regions of the ring than others (Figure 2B). This arises due to differences between the lateral and axial resolution achieved by 3D-SIM. Under our experimental conditions we calculated the limit of resolution in the lateral plane (*x*-*y* axis) to be 118 nm while in the axial plane (*z*-axis) it is 280 nm (refer to Materials and Methods). This means that in a rod-shaped cell that lies parallel to the microscope objective, only the top and bottom regions of the Z ring are imaged with the optimal resolution of 118 nm (see Figure 2B). Fortuitously, we noticed that fixed *B. subtilis* cells sometimes do not lie flat on the microscope coverslips (Figure 3A). As a result, the orientation of the Z ring is changed, moving closer towards the lateral plane where the optimal level of image resolution can be obtained in 3D-SIM. Importantly, when visualized under these conditions, the distribution of FtsZ is heterogeneous throughout the entire Z ring (Figure 3B).

To examine the level of heterogeneity of the Z ring in more detail, we took advantage of the fact that in spherical bacterial cells, such as *S. aureus*, Z rings will be orientated in all possible planes (Figure 3C). *S. aureus* is a human pathogen that is increasingly problematic in hospitals due to its ability to cause disease and develop resistance to antibiotics [34]. Furthermore, *S. aureus* FtsZ has been shown to be essential for cell viability [35] and lead compounds which show inhibitory action against FtsZ have been discovered [36],[37],[38]. However, the small size of *S. aureus* cells and their division in three different planes has made imaging of Z-ring structure and dynamics in vivo in this organism particularly challenging.

To examine the structure of the Z ring in live *S. aureus* cells, we utilized a strain ectopically expressing an FtsZ-GFP fusion from a low copy number plasmid [39]. In this strain, induction of FtsZ-GFP production with just 50 µM IPTG (isopropyl β-D-1-thiogalactopyranoside) had no detectable effect on cell growth or division [39]. Conventional microscopy on these cells confirmed that *S. aureus* Z rings could be detected readily in both the axial and lateral planes as predicted (Figure 3C). These Z rings appeared as smooth, uniformly stained structures by conventional optics. 3D-SIM, however, revealed a distinctly heterogeneous architecture for the *S. aureus* Z ring (Figure 3D). When imaged in the axial plane, *S. aureus* Z rings were almost identical to typical 3D-SIM images of the *B. subtilis* Z ring acquired in the same orientation (Figure 3E, Movie S2). Imaging in the lateral plane, however, confirmed that there was a heterogeneous, bead-like distribution of FtsZ throughout the entire Z ring (Figure 3D and 3F). Examination of the intensity plots of Z rings imaged in the lateral plane reveals that the concentration of FtsZ-GFP within the ring can typically vary by up to 4-fold (Figure 3G). In addition, visible “gaps” were often observed within the ring, similar to *B. subtilis*, which probably represent areas of the ring where little or no FtsZ is present. Restricting our analysis to Z rings imaged in the lateral (optimal) orientation, 26% of rings (n = 43) contained at least one visible gap. Measurements were also performed to examine the length and depth of the beads as shown schematically in Figure 3H. It was found that the majority of Z rings (80%; *n* = 43) had a bead length (measured along the circumference of the cell membrane) of 200 nm, with a maximum length of 400 nm. The maximum depth of the beads (measured across the axial plane of the bead; perpendicular to the cell membrane) was up to 200 nm. On average, the number of FtsZ beads per Z ring was 12±2 (SEM; standard error of the mean).

To ensure that this heterogeneous and bead-like localization pattern was not due to an *ftsZ-gfp* fusion being expressed in conjunction with native *ftsZ*, we visualized *S. aureus* Z rings in wild-type cells using immunofluorescence with anti-FtsZ antibodies raised against *B. subtilis* FtsZ [40]. Z rings visualized with anti-FtsZ antibodies showed an identical localization pattern to live cells (*n* = 113; compare Figure 3D and 3I). As expected, the number of FtsZ beads per Z ring observed with immunofluorescence was similar to rings observed in live-cell microscopy (10±2, compared to 12±2, respectively) and with similar dimensions. We noticed, however, that the number of cells containing at least one gap within the ring varied according to the concentration of antibody used. Using the highest antibody concentration tested (1∶50 dilution), 18% of cells contained a visible gap (*n* = 49), while 50% and 85% of the cells showed gaps when 1∶100 (*n* = 44) and 1∶10,000 (*n* = 20) dilutions were used, respectively. This is different to the 26% value obtained with live cells and likely reflects differences due to the fixation process and antibody concentrations [41]. However, the overall localization pattern of FtsZ is very similar in live and chemically fixed cells, indicating that the Z ring in *S. aureus* has a genuine bead-like arrangement.

### Z rings of *B. subtilis* and *S. aureus* Are Structurally Dynamic

Previous studies using FRAP have shown that there is a continual exchange of FtsZ molecules between the Z ring and the non-ring FtsZ pool throughout the entire lifetime of the Z ring [26],[27]. This rapid turnover is believed to allow a constant influx of FtsZ and GTP into the ring that is required for Z ring remodeling and constriction [26],[27]. However, these studies have been performed using conventional optics and do not reveal how the architecture of the Z ring is affected by this dynamic exchange. To address this issue we performed time-lapse using 3D-SIM. One major problem with all time-lapse studies is preventing photo-bleaching of the sample during the course of the experiment. This is particularly relevant with 3D-SIM, as decreasing signal-to-noise ratio with a sample over time will result in poor reconstructions. This is at least partially attenuated by the application of the new OMX Blaze technology. OMX Blaze allows minimal excitation energy to enter the sample by using light beam inferometry to create the structured pattern upon the sample and by utilizing the increased sensitivity and quantum efficiency of scientific CMOS (complementary metal oxide semiconductor) cameras as detectors (see Materials and Methods). This combination results in decreased image capture time and decreased photo damage to the living cells.

**Figure 4:**
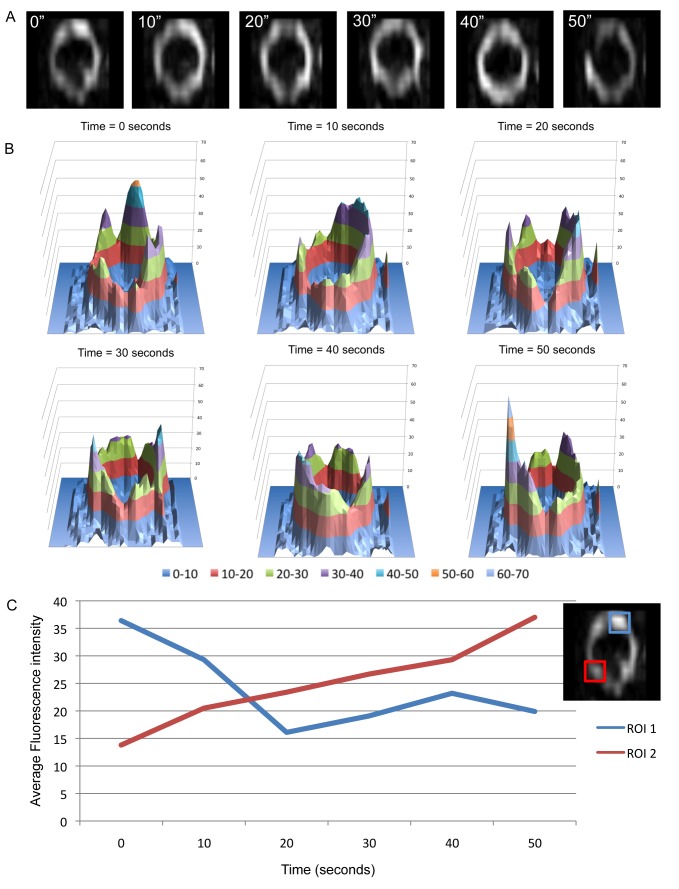
Time-lapse analysis of FtsZ localization within the Z rings of *B. subtilis* using 3D-SIM Blaze. (A) Changes in the distribution of FtsZ-GFP were clearly evident in the top and bottom regions of the Z ring when using 3D-SIM (OMX Blaze) in the *B. subtilis* strain SU570 grown in PAB at 30°C. Time (seconds) is indicated on the upper left corner of each image. Z ring diameter, 0.89 µm. (B) 3D intensity plots clearly show that the distribution of FtsZ in the Z ring remains heterogeneous and dynamic. Each graph represents an image for each time point as shown above. (C) To analyze FtsZ dynamics in more detail, two regions of interest were monitored over time showing how FtsZ-GFP fluorescence fluctuates in the Z ring.

In *B. subtilis*, we were able to obtain 3D-SIM time-lapse movies at 5- or 10-s time intervals over a 1-min period with OMX Blaze. A total of 77 Z rings were analyzed by this approach and Figure 4A shows a typical experiment in which a 3D-SIM image was captured every 10 s over a period of 1 min. The most noticeable feature of these time-lapse movies was how dramatically the fluorescence intensity of FtsZ-GFP dynamically changed within the Z ring (Movie S3). The distribution and position of FtsZ-GFP within the ring rapidly changed from one frame to the next (Movies S4 and S5). The diameter of the ring, however, tended to remain constant over the course of these 1 min movies, suggesting that active constriction itself did not occur within this time-scale in these cells. To illustrate the dynamic changes in FtsZ-GFP distribution around the Z ring, we generated 3D intensity plots that track differences in the position and intensity of fluorescence throughout the entire ring over time (Figure 4B). We also measured changes in FtsZ-GFP signal intensity in particular regions of interest (Figure 4C). Together these data demonstrate for the first time, to our knowledge, that the gross architecture of the Z ring, and not just the FtsZ molecules within this structure, is extremely dynamic and constantly changing.

**Figure 5:**
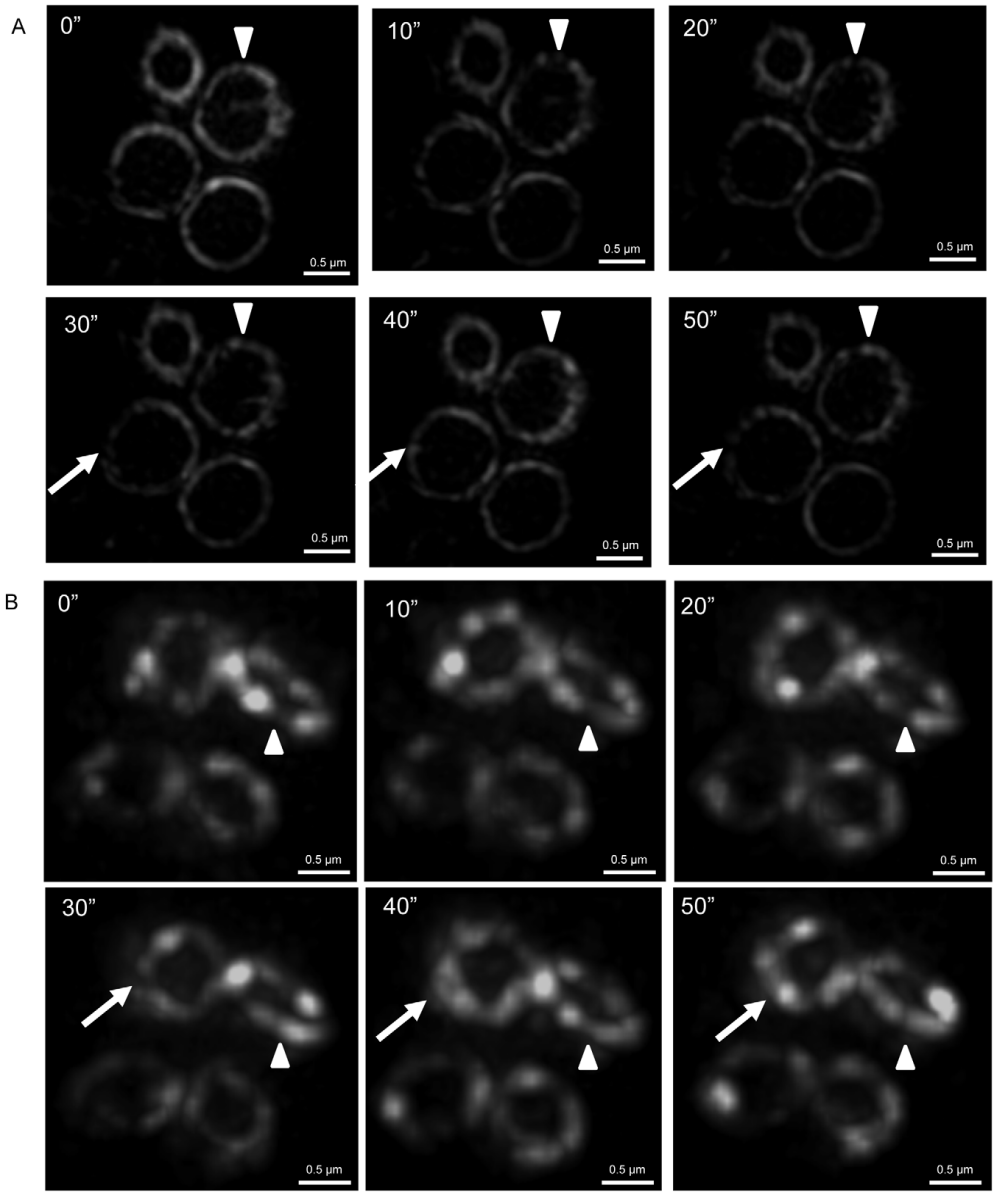
Z ring dynamics in *S. aureus*. (A) 3D-SIM (OMX Blaze) time-lapse images show how FtsZ localization changes within the Z ring in *S. aureus* RN4220 cells (SA89). A white arrowhead marks the position of a gap when it initially forms inside the Z ring. The subsequent position of the arrowhead in each time point does not change and indicates how FtsZ is redistributed to a region of the Z ring, which previously had very little FtsZ present. White arrows indicate the formation of additional gaps in the Z ring. (B) Deconvolution time-lapse microscopy of SA94 cells expressing FtsZ-GFP. Time (s) is indicated on the upper left-hand side for each image. Cells were grown in L-broth at 37°C in the presence of 0.05 mM IPTG.

Next we attempted to establish whether *S. aureus* FtsZ undergoes a similar dynamic movement to that of *B. subtilis*. To do this, *S. aureus* SH1000 cells expressing FtsZ-GFP were visualized using 3D-SIM (with OMX Blaze) at 10-s time intervals for 50 s. However, unlike *B. subtilis*, visualization of the Z ring at later time points (30–50 s onwards) in *S. aureus* SH1000 was hampered by extensive bleaching of the GFP signal (unpublished data). We were, however, able to obtain images of *S. aureus* RN4220 cells expressing FtsZ-GFP [39]. 3D-SIM images taken every 10 s for 50 s showed changes to the heterogeneity of the ring similar to those seen with *B. subtilis* FtsZ (see arrowheads and arrows in Figure 5A; see also Movie S6). Indeed, using conventional wide-field deconvolution time-lapse microscopy of the SH1000 strain of *S. aureus*, we were still able to observe differences in FtsZ-GFP fluorescence intensity within the Z ring similar to those seen with 3D-SIM as shown in Figure 5B and Movie S7. This confirms that the overall localization pattern of FtsZ is similar in both *S. aureus* backgrounds. The architectural changes seen with 3D-SIM and conventional wide-field deconvolution fluorescence are consistent and similar to the dynamic changes in FtsZ localization seen with *B. subtilis* Z rings.

Although we also observed slight movement of individual cells during the length of the movies, it is unlikely that this movement explains the change in ring heterogeneity over time because each ring taken at the different time intervals shows a completely different architecture (i.e., images of the ring are non-super imposable through rotation). Therefore, it is unlikely that the changes in Z ring structure are due to the rotation of the ring around its axial plane caused by passive movement of the cells and are more likely due to remodeling of FtsZ.

### Z ring Dynamics Occurs Independently of Constriction

To fully understand how the Z ring functions in division, we need to understand how FtsZ is distributed within the Z ring and how the ring architecture changes during the process of constriction. While all Z rings appeared dynamic in our initial time-lapse experiments described above, these experiments were performed over a short time period in which it was not possible to see visible constriction. For this reason, we then carried out longer 3D-SIM time-lapse experiments over a period of 10 min (using a longer interval of 1 min between images) in an attempt to capture the constriction process and to determine if FtsZ dynamics differ between constricting versus non-constricting Z rings in *B. subtilis*. Indeed, we were able to visualize Z ring constriction in some cells using this approach (*n* = 25). Interestingly, all Z rings (*n* = 99) showed a similar dynamic redistribution of FtsZ in these experiments, including those that were undergoing active constriction (Movies S8 and S9). This suggests that the Z ring is structurally dynamic both before and during constriction.

However, to establish more conclusively whether FtsZ dynamics within the ring can occur completely independently of Z ring constriction, we used the *B. subtilis* temperature-sensitive mutant, *div-355*, which contains a mutation in the essential division gene *divIC* and produces Z rings that do not constrict at high temperatures. DivIC is a late cell division protein, which forms a complex with other cell division proteins including FtsL, DivIB, and PBP2B [4],[42],[43],[44], and their recruitment to the division site requires FtsZ. PL91 cells (*div-355* mutant) divide normally at the permissive temperature of 30°C, but at 45°C, Z rings do not constrict and division is completely inhibited, producing filamentous cells with no division septa [45].

**Figure 6:**
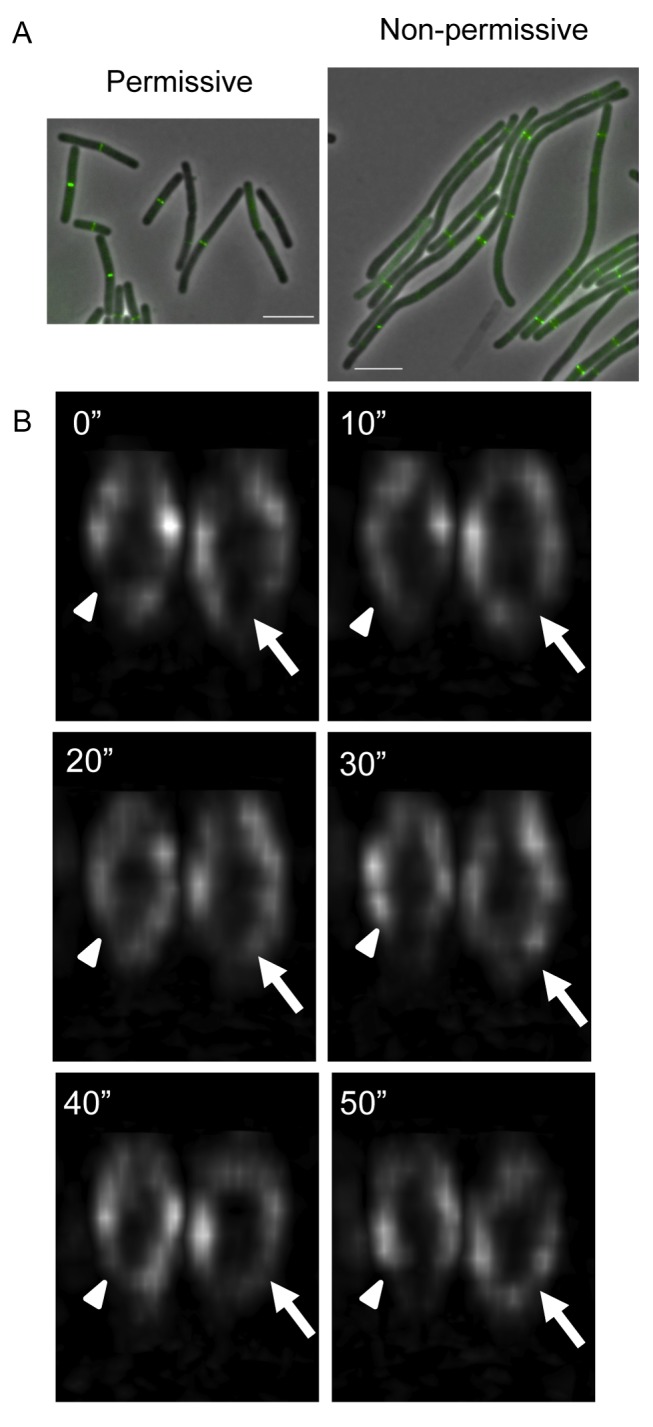
Monitoring FtsZ dynamics in non-dividing *B. subtilis* cells. (A) At the permissive temperature SU744 (*div-355*) cells are able to divide, but at the non-permissive temperature division is inhibited as seen by the increased cell length. FtsZ-GFP was induced with 0.005 mM IPTG and grown in PAB at 30°C until mid-exponential growth before dilution and further growth at 45°C for 30 min to inhibit division. Scale bar, 5 µm. (B) To monitor FtsZ dynamics in non-dividing cells we visualized FtsZ-GFP localization in cells with the *div-355* mutation at non-permissive temperature using conventional deconvolved microscopy (images acquired on the OMX Blaze system). FtsZ remains dynamic even when division is inhibited and Z ring constriction does not occur. A white arrowhead marks the position of a gap when it initially forms inside the Z ring. White arrows indicate the formation of additional gaps in the Z ring. Time (s) is indicated in the upper left corner. Z ring diameter, ∼0.9 µm.

To visualize FtsZ dynamics at the non-permissive temperature, we could not use the single copy FtsZ-GFP fusion protein because it is not functional at 45°C. Instead we used an inducible second copy of the FtsZ-GFP fusion protein, which co-localizes with native FtsZ. Low level expression of the fusion protein in SU568 (*amyE::*P*spac-ftsZ-gfp*) cells also revealed a heterogeneous distribution of FtsZ-GFP inside the Z ring without perturbing cell division (unpublished data). The fusion protein was susceptible to photo-bleaching during time-lapse experiments even with OMX Blaze in 3D-SIM mode, presumably because of the low abundance of the fusion protein in the cell (unpublished data). Therefore, conventional wide-field fluorescence time-lapse experiments were performed. The superior optics on the OMX microscope produce a 3D deconvolved image with improved signal-to-noise ratio when compared to the raw conventional image (refer to Materials and Methods). Deconvolved time-lapse movies of SU568 cells showed that FtsZ-GFP, when expressed as a second copy, undergoes dynamic changes in the Z ring that closely resembled SU570 cells at 37°C (Movie S10). Furthermore, FtsZ-GFP inside the Z ring appeared heterogeneous and dynamic when SU568 cells were grown at 45°C and divided normally (Movie S11). Next the FtsZ-GFP construct was introduced into cells harboring the *div-355* mutation to create strain SU744 (*div-355 amyE::*P*spac-ftsZ-gfp*). This strain was used to examine whether FtsZ remains dynamic in cells in which Z ring constriction and division are inhibited. Growth and division in SU744 cells at the permissive temperature was normal as previously reported and ∼36% of Z rings appeared to be in the process of constriction (*n* = 115; Figure 6A, left panel). However, when these cells were grown at the non-permissive temperature, the cells became filamentous and the percentage of constricting Z rings decreased to ∼2% (*n* = 125; Figure 6A, right panel). FtsZ-GFP distribution in the Z ring was heterogeneous and dynamic at the permissive temperature in SU744 cells. However, there was no apparent effect on FtsZ-GFP distribution inside the Z ring or its dynamics when division was inhibited at the non-permissive temperature (Figure 6B and Movie S12). These results confirm that the Z ring remains structurally dynamic even in the complete absence of Z ring constriction.

### EzrA and PBP2 Localization Are Heterogeneous and Dynamic in *S. aureus*

The heterogeneous structure of the Z ring revealed by 3D-SIM raises the question of how other division proteins are arranged at the division site. To address this question, we examined the localization pattern of two divisome proteins: EzrA and PBP2 (penicillin binding protein-2). In *B. subtilis*, EzrA is recruited to the Z ring early in an FtsZ-dependent manner. EzrA is an important regulator of Z ring formation and promotes the efficient recruitment of PBP1 to the divisome [33],[46],[47]. In *S. aureus*, EzrA interacts with almost all the known cell division proteins in a BACTH assay, indicating that EzrA is a central component of the *S. aureus* divisome [48],[49].

**Figure 7:**
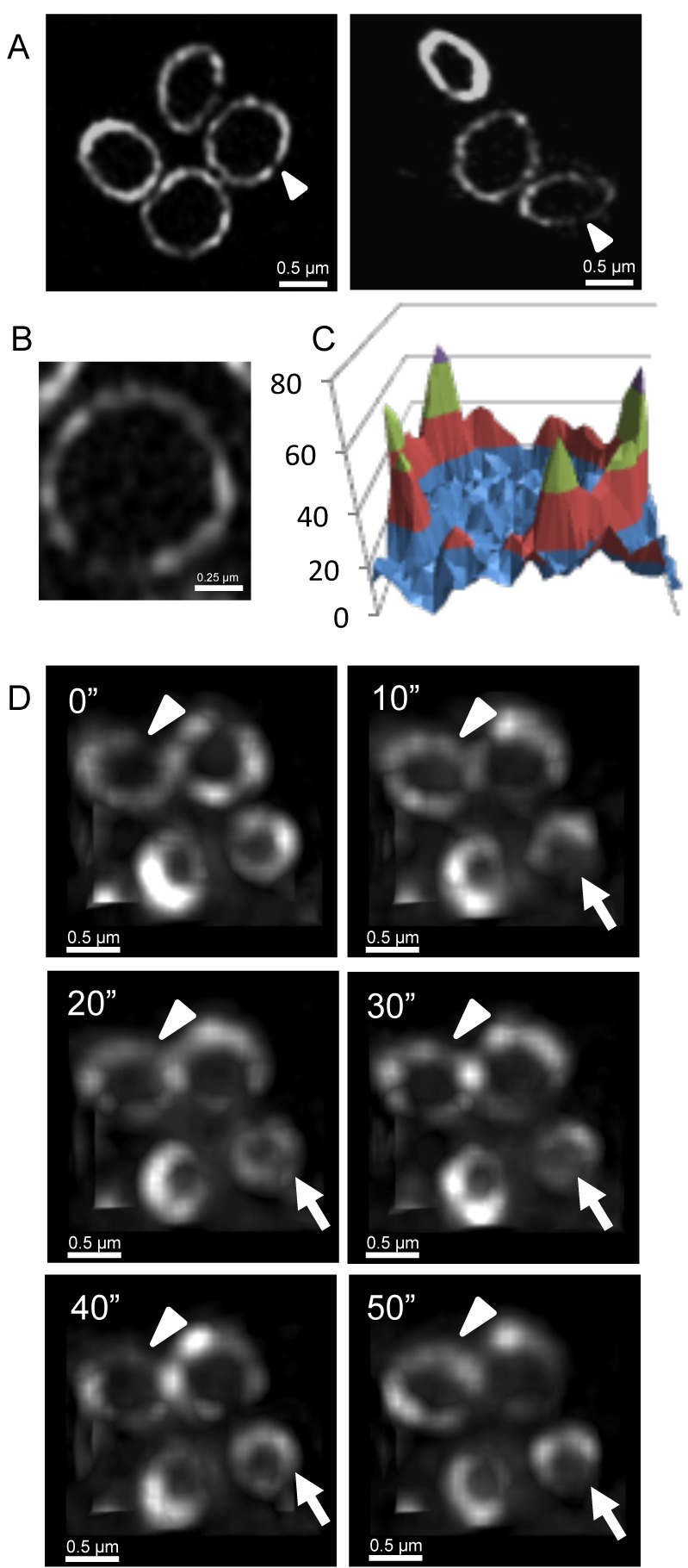
EzrA rings in *S. aureus* are also heterogeneous and show dynamic movement. (A) To examine EzrA-GFP localization using 3D-SIM (OMX V3), *S. aureus* strain SA126 was grown in L-broth at 37°C. The EzrA-GFP fusion protein displays a similar type of localization at the division site as FtsZ. (B) A close-up image of an EzrA ring in SA126 cells imaged in the lateral orientation with 3D-SIM (OMX V3). (C) A 3D intensity plot of EzrA rings orientated in the lateral plane shows a similar profile to FtsZ-GFP. (D) Deconvolved images were obtained through conventional wide-field fluorescence to monitor the localization of EzrA-GFP over time (acquired using OMX Blaze system). EzrA-GFP localization is dynamic and similar to that observed using FtsZ-GFP in *S. aureus*. White arrowheads show areas where the EzrA concentration is reduced. Arrowheads indicate the formation of additional gaps in the EzrA-GFP rings. Time (s) is indicated on the upper left-hand side for each image.

We used 3D-SIM to visualize EzrA localization in *S. aureus* cells in which the wild-type *ezrA* gene is replaced by a functional *ezrA-gfp* fusion expressed from its native promoter [48]. EzrA-GFP in live cells localized in a bead-like pattern that appeared identical to that of FtsZ (Figure 7A and 7B). Fluorescence intensity quantification of the EzrA ring showed distinct peaks and noticeable gaps within the ring (Figure 7C). Similar to FtsZ in the Z ring, the average number of EzrA beads per ring was about 13±2 SEM in all the cells that were scored (*n* = 43), with most of the beads (83%) measuring 200 nm in length. About 23% of the EzrA rings observed showed at least one visible gap within them, which is very similar to the number of gaps observed in Z rings in live *S. aureus* cells (26%). Time-lapse microscopy of EzrA-GFP in *S. aureus* using conventional wide-field fluorescence microscopy and deconvolution (performed under the same conditions used for FtsZ-GFP) showed EzrA to have the same dynamic nature as FtsZ-GFP, with changes to the overall heterogeneity of the ring over time in all the cells observed (*n* = 50) (Figure 7D; Movie S13).

**Figure 8:**
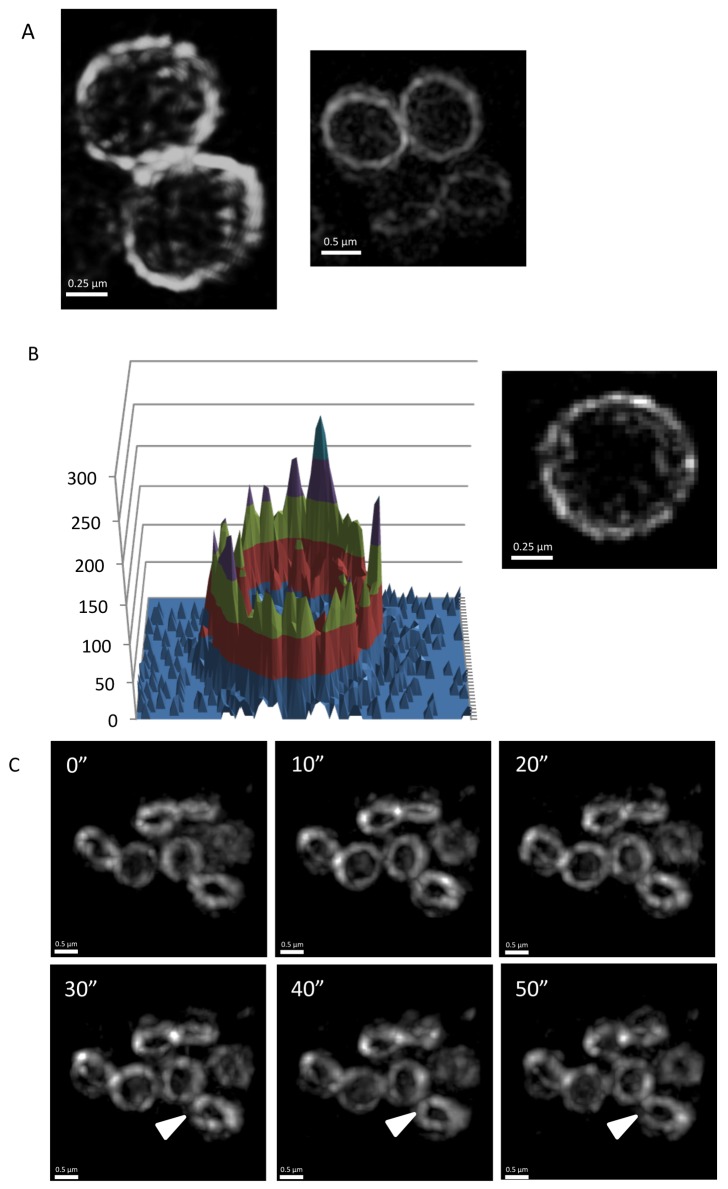
PBP2 rings in *S. aureus* are also heterogeneous and show dynamic movement. (A) To examine GFP-PBP2 localization using 3D-SIM (OMX V3), *S. aureus* strain SA136 was grown in L-broth at 37°C. (B) A 3D intensity plot of a GFP-PBP2 ring orientated in the lateral plane shows a similar profile to FtsZ-GFP (left) and a close-up image of a PBP2 ring (right) in SA136 cells imaged in the lateral orientation with 3D-SIM (OMX V3) (C) Deconvolved images were obtained through conventional wide-field fluorescence microscopy; the localization of GFP-PBP2 over time is dynamic and similar to that observed using FtsZ-GFP. Arrowheads indicate the formation of additional gaps in the PBP2 rings. Images were acquired on the OMX Blaze system. Time (s) is indicated on the upper left-hand side for each image.

PBP2 is an essential protein in *S. aureus* that catalyzes the final stages of peptidoglycan synthesis during the formation of the division septum. It does not interact with *S. aureus* FtsZ in a BACTH assay [50] but is recruited to the division site possibly through interaction with other divisome components as well as through substrate recognition [51]. We used an N-terminal GFP-PBP2 fusion [51] that was expressed through a xylose-inducible promoter to determine its localization and dynamics. Like FtsZ and EzrA, 3D-SIM images of PBP2 rings also showed a heterogeneous structure (Figure 8A and 8B). Furthermore, conventional wide-field deconvolution time-lapse microscopy of GFP-PBP2 displayed a similar dynamic movement in all cells examined (*n* = 50; Figure 8C and Movie S14).

Taken together, *S. aureus* FtsZ, EzrA, and PBP2 all show a very similar heterogeneous localization pattern. It is therefore tempting to speculate that the entire divisome in *S. aureus* is heterogeneous and dynamic.

## Discussion

In this work, we visualized the Z ring using 3D-SIM to further understand how FtsZ is localized within this structure and subsequently how it constricts to allow cytokinesis in bacteria. Previous attempts to address this issue using different microscopy techniques have been hampered by the inability to visualize the complete 3D architecture of the Z ring and examine how it is dynamically remodeled in live cells. We were able to overcome this problem through the use of 3D-SIM, as this form of super resolution microscopy breaks the diffraction barrier of resolution in both lateral and axial planes. We found that the Z ring is composed of a heterogeneous distribution of FtsZ in a bead-like arrangement in both *B. subtilis* and *S. aureus*. Moreover, we used OMX Blaze technology to show that this bead-like distribution undergoes constant changes both before and during Z ring constriction. This has important implications for understanding constriction (see below), and also shows that the Z ring is not a fixed “scaffold” upon which FtsZ molecules can move in and out. Rather, dynamic turnover of the Z ring additionally involves gross changes in the organization of the ring. Lastly, we examined other components of the divisome and found that they also exhibit a dynamic bead-like localization pattern remarkably similar to FtsZ.

### The 3D Architecture of the Z Ring

For decades conventional fluorescence microscopy has depicted the Z ring as a smooth cable-like structure, hinting that the ring is composed of a single continuous polymer that wraps around the cell [52]. More recently, electron cryotomography (ECT) of *C. crescentus* cells provided evidence for a very different Z ring structure, showing the ring to be composed of just a few short FtsZ filaments with a sparse and discontinuous arrangement at the division site [19]. While ECT offers a large increase in resolution over conventional light microscopy, the relatively low number of FtsZ protofilaments observed using this technique, coupled with the inability to label FtsZ directly, raises concerns about the sensitivity in detecting total FtsZ present within the Z ring.

Using super resolution 3D-SIM microscopy in live cells of *B. subtilis* and *S. aureus* labeled with FtsZ-GFP as well as in fixed cells with immunofluorescence, we now show that FtsZ is distributed in a heterogeneous, bead-like pattern around the entire Z ring. This confirms that the Z ring is not a homogenous band of FtsZ, but rather it contains regions of high FtsZ abundance and regions of little or no FtsZ protein. Interestingly, the lengths of the beads we measured in the Z ring (average bead length 200 nm and maximum length 400 nm) correlate quite closely with the lengths of FtsZ protofilaments typically observed in vitro (50–300 nm), suggesting that the beads may be composed of short FtsZ filaments similar to those detected by ECT [13],[53]. In addition, we visualized gaps in the Z ring that hint at a discontinuous structure. We cannot state unequivocally that the ring is discontinuous because 3D-SIM is not capable of single molecule detection. However, given the large size of these gaps (at least 118 nm), and taking into account that every FtsZ molecule is labeled with GFP in some of our strains, we believe it is unlikely that a continuous FtsZ polymer strand spanning this entire region would fail to be detected by 3D-SIM.

**Figure 9:**
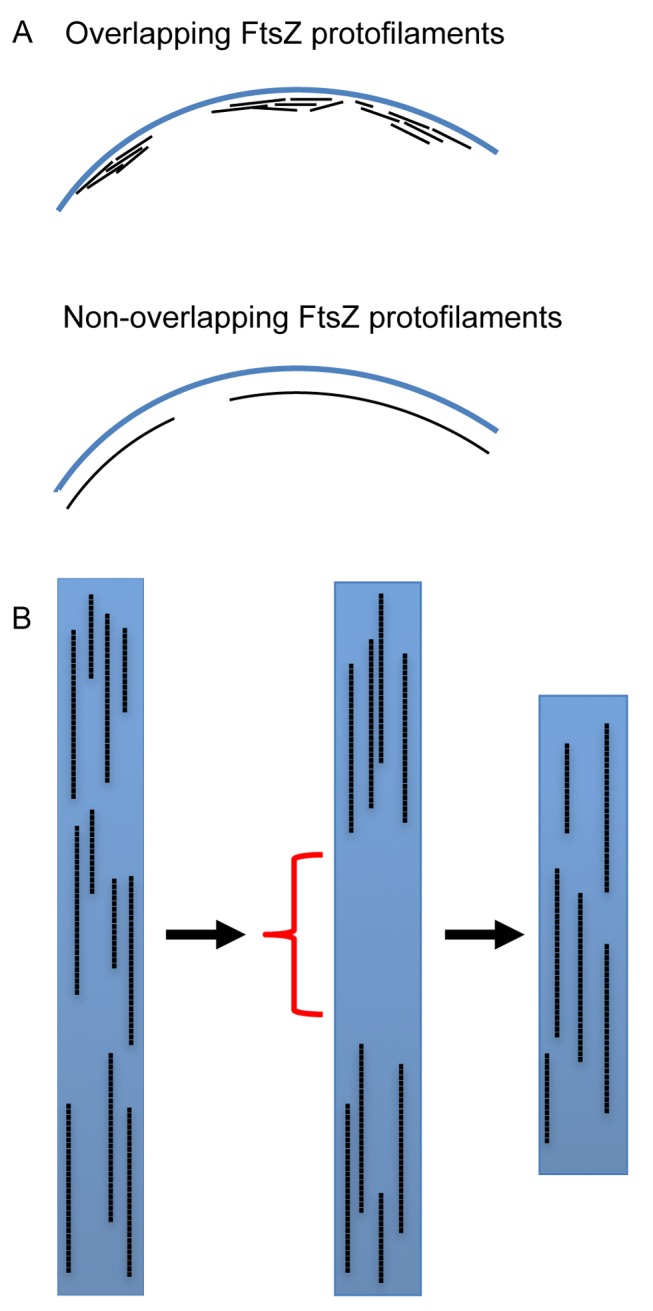
Models for the arrangement of FtsZ protofilaments inside the Z ring. (A) We propose two models to describe the architecture of the Z ring. The overlapping model predicts that short FtsZ protofilaments begin to bundle in both lateral (circumferential) and radial directions of the cell. Alternatively, FtsZ protofilaments could be arranged into a single layer in the radial direction and only bundle in the lateral direction. (B) The amount of FtsZ inside the Z ring is constant throughout constriction, but the distribution of FtsZ inside the rings fluctuates over time. As constriction begins the Z ring exerts a localized pinching force on the membrane where increased levels of FtsZ are found. Gaps (indicated by red brackets) in the Z ring allow constriction to occur continuously as the circumference of this structure becomes reduced (indicated by the decrease in height of the blue rectangle).

Combining new insights from 3D-SIM with previously published data we propose two models to account for how FtsZ is distributed inside the Z ring and this is illustrated in Figure 9A. Both models predict that the Z ring is composed of a heterogeneous arrangement of short FtsZ protofilaments around the inner cell membrane. Some regions of the Z ring (beads) contain a high density of FtsZ protofilaments that are distributed around the inner cell membrane in a heterogeneous fashion, while others (gaps) contain little or no FtsZ. In the “overlapping” model (Figure 9A, left panel), short FtsZ protofilaments on the membrane are loosely bundled with radial thickness (out from the membrane towards the cell center) as well as along the cell membrane as suggested by PALM imaging [31]. In the second “non-overlapping” model for Z ring architecture, the short FtsZ protofilaments are distributed on the membrane with only a single layer in the radial direction (Figure 9A, right panel). We favor the non-overlapping model for Z ring architecture because EM techniques like ECT are currently the only way to visualize individual FtsZ protofilaments and always depict FtsZ in a single, radial layer around the inner cell membrane, even when it is overproduced [19].

### Implications for Generating a Contractile Force

What role does the architecture of the Z ring have in the process of constriction? Previously, a model was proposed to describe how discontinuous Z rings might generate a contractile force based on ECT images of the Z ring in *C. crescentus* [19]. Known as the iterative pinching model, it was suggested that FtsZ undergoes continual cycles of polymerization (forming short linear filaments on the membrane), polymer bending (generating a localized inward force), and disassembly from the membrane. Over many of these cycles, enough force is generated to pull in the cytoplasmic membrane and facilitate cell division. A central idea of this model is that FtsZ is continually redistributed around the Z ring. However, this could not be tested by the ECT technique as it requires samples to be fixed. Using OMX Blaze technology, we were able to observe the structural dynamics of FtsZ localization in live cells by 3D-SIM, on a time scale of as little as 5 s. This showed directly that FtsZ is indeed rapidly redistributed within the Z ring during constriction, and is fully consistent with an “iterative pinching” type mode of constriction.

The gaps observed in the Z ring by 3D-SIM might also serve an important purpose during constriction. In a recent study it was predicted that continual constriction of the Z ring may require the generation of gaps in the ring through turnover of FtsZ. This was based on the observation that Z rings, assembled in vitro in tubular liposomes, could only constrict to a small extent before quickly halting in the absence of GTP hydrolysis [54]. This presumably occurred because additional gaps within the Z ring could not be generated to accommodate the structural changes that occur in the Z ring during constriction. Our results with 3D-SIM now confirm the existence of gap regions in the Z ring containing little or no FtsZ.

Taken together, it appears that the gaps and beads we observe in the Z ring may serve different roles required for the continual constriction of the ring (Figure 9B). The beads most likely represent regions where increased amounts of FtsZ generate a localized contractile force on the inner membrane, while the gaps represent areas that allow the Z ring to continually reduce its circumference as it pulls in the cell envelope. This may explain how a heterogeneous arrangement of FtsZ can provide an equal contractile force on the inner cell membrane over time.

### Coordination between Early and Late Cell Division Proteins Triggers Z Ring Constriction?

Interestingly, our results demonstrate that the Z ring is heterogeneous and structurally dynamic throughout its entire lifetime, not just during constriction. Moreover, these same structural dynamics are seen in a mutant that is unable to undergo Z ring constriction at all. This indicates that while FtsZ dynamics are likely to play an important role in the constriction process (see above), these dynamics alone are not sufficient to initiate constriction. Rather, an additional factor must be required to trigger the constriction process and allow cytokinesis at the appropriate time.

What might this trigger be? One idea is that the signal to constrict may be provided by later acting cell division proteins, which connect directly to FtsZ via the divisome. These proteins also interact with the cell wall synthesis machinery and could function to coordinate Z ring constriction with septal peptidoglycan production. In support of this idea, previous studies have shown that the inactivation of one or more components of the divisome results in an inhibition of Z ring constriction [55],[56],[57]. Indeed, we found in the present study that DivIC inactivation caused a defect in Z ring constriction without affecting the structure or dynamics of the ring. Under these conditions the Z ring appears ready to commit itself to constrict, but cannot go ahead until the divisome properly assembles.

Presumably, to properly coordinate Z ring constriction and cell wall synthesis direct interactions must be maintained between all necessary components of the divisome throughout the cell division process. Consistent with this idea we found that EzrA and PBP2 localization closely resembled FtsZ in both structure and dynamics. This suggests that bacterial cell division is mediated by a heterogeneous and highly dynamic multi-protein machine.

## Materials and Methods

### Bacterial Strains and Growth Conditions

See Table 1 for a list of the strains used in this study. To construct strain SA126, JGL227 was transduced into the ELC2 (protein A-deficient NCTC 8325 *S. aureus* strain) background using φ11 phage transduction as previously described [58]. In all experiments bacterial cells were harvested and analyzed at the mid-exponential phase of growth. All *B. subtilis* strains were grown in Penassay broth (1.75% Bacto antibiotic medium 3) or Spizizen minimal medium (0.5% w/v glucose, 0.02% w/v MgSO4, 1× mineral salts A) [59] supplemented with L-tryptophan (50 µg ml^−1^), casamino acids (0.05% w/v), and 1× trace metals [25] in the absence of antibiotics with vigorous shaking. IPTG was added to 0.005 mM where appropriate. SU570 cells were grown and imaged at the permissive temperature of 30°C [33]. Cells with the *div-355* mutation were grown at the permissive temperature of 30°C, or to inhibit DivIC function, these cells were transiently grown at the non-permissive temperature of 45°C for 30 min before imaging [45]. All *S. aureus* strains were cultured at 37°C in L-broth with 2% glucose and, where appropriate, 5 µg erythromycin ml^−1^, 25 µg lincomycin ml^−1^, 10 µg chloramphenicol ml^−1^, and 0.05 mM of IPTG. Total cellular FtsZ levels (FtsZ-GFP and native FtsZ) in *S. aureus* cells grown with this concentration of IPTG have previously been shown to be approximately 2-fold higher relative to wild-type *S. aureus* cells [39]. However, no growth or cell division defects were associated with this level of FtsZ overproduction.

### Live Cell Microscopy

*B. subtilis* and *S. aureus* cells were prepared for live cell microscopy as described previously [25] on 2% (w/v) agarose pad containing the same media used for growth. The agarose pad was then inverted so that the cell suspension is in contact with the coverslip of a Fluorodish (refractive index: 1.525).

### Immunofluorescence

Immunofluorescence of *B. subtilis* cells was performed as previously described [25], with the exception that cells were adhered to a poly-L-lysine-treated Fluorodish (World Precision Instruments) and treated with lysozyme. Cells were fixed in 10 ml of ice-cold (−20°C) methanol for 1 h prior to lysozyme treatment. Anti-FtsZ antibodies (raised against *B. subtilis* FtsZ in rabbits) were added at different concentrations and incubated overnight at 4°C. These antibodies react specifically against *S. aureus* and *B. subtilis* FtsZ and therefore provide an accurate localization pattern of FtsZ in both organisms [35],[39]. Secondary antibody (goat anti-rabbit IgG conjugated to Alexa488; Sigma) was added at a dilution of 1∶100 and the cells were then mounted in Vectashield (Vector Laboratories) mounting medium. Immunofluorescence of *S. aureus* cells was performed as previously described [35] except that a gentle lysis was performed using lysostaphin (Sigma) at a final concentration of 30 ng ml^−1^ for 30 s before the addition of the anti-FtsZ primary rabbit antibody, a Fluorodish was used, and cells were mounted in Vectashield.

### Conventional Fluorescence Microscopy Using the Zeiss Axioplan 2 Microscope

Conventional (wide-field) fluorescence microscopy was performed using a Zeiss Axioplan 2 microscope (Carl Zeiss) as described previously [25]. In *B. subtilis*, FtsZ-GFP and the cell membrane were co-visualized with FM 4-64 (Invitrogen) at a final concentration of 1 µg ml^−1^. GFP and FM 4-64 fluorescence were visualized with filter sets 488009 and 488015 (Carl Zeiss), respectively.

### Conventional Wide-Field Deconvolution Fluorescence Microscopy and 3D-SIM Super-Resolution Microscopy using the Deltavision OMX Imaging Systems

Both conventional fluorescence microscopy and three dimensional-structured illumination microscopy (3D-SIM) were implemented on two versions of the DeltaVision OMX imaging system (Applied Precision Inc, Issaquah, USA): the OMX V3 and a new system known as OMX Blaze (Applied Precision, a GE Healthcare Company).

The DeltaVision OMX V3, based on the system described in [32],[60] uses solid state multimode lasers (488, 593 nm) to provide wide-field illumination and multi-channel images that are captured simultaneously using two Photometrics Cascade (Photometrics, Tucson, USA) back-illuminated EMCCD cameras (>90% QE) with a 512×512 CCD, and on-chip charge multiplication. Data capture used an Olympus UPlanSApo 100× 1.4 NA oil objective and standard excitation and emission filter sets (in nm, 488 EX/500–550 EM and 592.5 EX/608–648 EM). 3D-SIM images were sectioned using a 125 nm z-step.

The new Deltavision OMX Blaze allows ultra-high speed illumination and acquisition. A full description of this new system follows. Laser excitation light (488 nm and 592 nm) was shuttered using a high speed tilt mirror with open times and close times of 0.2 ms. The laser excitation light was subsequently coupled into a broadband single mode optical fiber. Light exiting the fiber was first split into three beams, then passed through separate modules to control the phase and angle of the three beam pattern, and finally focused downstream at the back focal plane of the objective lens to generate a 3D interference pattern at the sample plane. Within the phase control module, each of the two outer beams was directed to propagate through a separate pair of windows with individual tilt control. By tilting a given window pair in complementary directions (to cancel the lateral refractive beam translation), a path length change was imparted on the respective outer beam to shift the phase of the interference pattern at the sample plane. Phase shifts of the interference pattern were completed in 0.2 ms. Within the angle control module, a tilt mirror was employed to direct the three beam pattern to one of three mirror clusters, each of which imparted a distinct rotation to the three-beam pattern. The beam pattern from each of the three rotation paths was redirected back to a common exit path by reflecting a second time from the tilt mirror. Angle shifts of the interference pattern were completed in 4 ms. Images were captured on two PCO Edge scientific CMOS cameras (each dedicated to a specific channel) with acquisition rates of up to 400 fps, using an Olympus PlanApo N 60× 1.42 NA oil objective and excitation and emission filters as above. Unprocessed image stacks were composed of 15 images per z-section (five 72 degree phase-shifted images per angle at each of three interference pattern angles, +60, 0, and −60 degrees). The z-sections were completed at a spacing of every 125 nm for a total raw data image count of 120 images per 1 µm sample z-stack. Full super-resolution 1 µm thick image stacks with 40×40 µm field of view (512×512 pixels unprocessed image size with 16 bit dynamic range) could be captured with a total acquisition time of 1 s. The output of raw data from the OMX Blaze was processed as for OMX V3 data. The microscope is routinely calibrated to calculate both the lateral and axial limits of image resolution under our experimental conditions [32],[60]. Reconstructed images were rendered in 3D, with interpolation, using Imaris version 7.0.0 (Bitplane Scientific).

Both the OMX V3 and OMX Blaze systems were used in this study to capture conventional wide-field fluorescence images [61]. Immunofluorescence microscopy was performed using the Deltavision OMX V3 imaging system in 3D-SIM mode. Live cell time-lapse fluorescence microscopy was performed using both OMX V3 and OMX Blaze systems, either in the conventional or 3D-SIM mode.

### Image Analysis

For 3D-SIM OMX V3 images, time series were compiled from single time points using Imaris software version 7.0.0 (Bitplane Scientific) to create a time-lapse series. The fluorescence at each time point was normalized. All raw images obtained in conventional mode were deconvolved using a constrained iterative algorithm (SoftWorX 4.0, Applied Precision Inc). This algorithm has been quantitatively verified [62] to accurately represent the original 3D object. All 3D-SIM and conventional mode images were analyzed using Imaris version 7.0.0 (Bitplane Scientific). For clarity of display, small changes to brightness and contrast were performed on 3D reconstructions. Only linear changes were made to brightness and contrast of the images. Non-linear (gamma setting) changes to the images were not performed. 3D intensity plots were generated using data obtained through the data inspector tool in SoftWorX. The total sum of fluorescence within the Z ring was quantified in Imaris.

## Supporting Information

Figure S1 Non-constricting and constricting Z ring appearance in live cells of SU570 using 3D-SIM (OMX V3). Visualizing the heterogeneous distribution of FtsZ-GFP is easier in Z rings with a diameter between ∼0.3 and 0.9 µm. Constricting Z rings (white arrow) with a diameter less than 0.3 µm are harder to visualize any heterogeneous distribution of FtsZ-GFP.
(TIF)

Figure S2 Immunofluorescence labeling of wild-type *B. subtilis* cells (SU5) reveals a similar heterogeneous distribution of FtsZ inside the Z ring. (Ai–Aii) 1∶100 dilution of anti-FtsZ. Z ring diameter, 0.9 µm (Bi–Bii) 1∶10,000 dilution of anti-FtsZ. Z ring diameter, 0.7 µm and 0.5 µm, respectively. Cells were grown at 30°C in PAB and imaged using 3D-SIM (OMX V3).
(TIF)

Figure S3 Additional examples of 3D intensity plots of *B. subtilis* Z rings. See Figure 4. SU570 cells were imaged using 3D-SIM (OMX V3).
(TIF)

Movie S1 The Z ring in *B. subtilis* can be rotated around the *z*-axis to view how FtsZ-GFP is distributed around the ring in SU570. Cells were grown at 30°C in PAB and imaged using 3D-SIM (OMX V3).
(MPG)

Movie S2 The Z ring is also heterogeneous in *S. aureus* and can be viewed in different orientations. SA98 cells were grown in L-broth with 0.05 mM IPTG and imaged using 3D-SIM (OMX V3).
(MOV)

Movie S3 Field of view of SU570 cells grown in PAB at 30°C imaged using OMX Blaze. One image was acquired every 5 s over a period of 1 min.
(AVI)

Movie S4 3D-SIM (OMX Blaze) time-lapse movies in *B. subtilis* show FtsZ-GFP dynamically changes its localization within the Z ring in SU570. One image was acquired every 5 s over a period of 1 min.
(AVI)

Movie S5 3D-SIM (OMX Blaze) time-lapse movie of a constricting Z ring in *B. subtilis* reveals dynamics continue throughout division in strain SU570. One image was acquired every 5 s over a period of 1 min.
(AVI)

Movie S6 Visualization of Z ring dynamics in *S. aureus* using 3D-SIM (OMX Blaze) in SA98 cells induced with 0.05 mM IPTG. One image was acquired every 10 s over a period of 1 min.
(WMV)

Movie S7 Visualization of Z ring dynamics in *S. aureus* using conventional deconvolved time-lapse microscopy. One image was acquired every 10 s over a period of 1 min. SA98 cells were grown in L-broth with 0.05 mM IPTG and imaged on the OMX Blaze system.
(WMV)

Movie S8 Visualization of Z ring dynamics in a non-constricting *B. subtilis* Z ring over 10 min. A 3D-SIM image was acquired every minute over a period of 10 min. SU570 cells were grown in PAB at 30°C. Images acquired using 3D-SIM (OMX V3).
(MOV)

Movie S9 Visualization of Z ring dynamics in a constricting *B. subtilis* Z ring over 10 min. A 3D-SIM image was acquired every minute over a period of 10 min. SU570 cells were grown in PAB at 30°C. Images acquired using 3D-SIM (OMX V3).
(MOV)

Movie S10 Conventional wide-field time-lapse movie of SU568 cells grown in PAB at 37°C with 0.005 mM IPTG. Similar to SU570 cells the distribution of a second copy FtsZ-GFP in the Z ring is heterogeneous and dynamic. One image was acquired every 10 s over a period of 1 min. Images acquired using OMX Blaze.
(AVI)

Movie S11 Conventional wide-field time-lapse movie of SU568 cells grown in PAB at 45°C with 0.005 mM IPTG. Growth at higher temperatures does not affect Z ring structure or dynamics. One image was acquired every 10 s over a period of 1 min. Images acquired using OMX Blaze.
(AVI)

Movie S12 Conventional deconvolved time-lapse microscopy of SU744 cells at the non-permissive temperature of 45°C. FtsZ remains dynamic even in non-dividing *B. subtilis* cells. One image was acquired every 10 s over a period of 1 min. Images acquired using OMX Blaze.
(AVI)

Movie S13 EzrA-GFP rings are also dynamic in *S. aureus*. One image was acquired every 10 s over a period of 1 min using conventional deconvolved time-lapse microscopy. SA126 cells were grown in L-broth at 37°C. Images acquired using OMX Blaze.
(AVI)

Movie S14 GFP-PBP2 rings are dynamic and similar to FtsZ and EzrA. One image was acquired every 10 s over a period of 1 min using conventional deconvolved time-lapse microscopy. SA136 cells were grown in L-broth at 37°C. Images acquired using OMX Blaze.
(WMV)

## Abbreviations

BACTH: bacterial two-hybrid
CMOS: complementary metal oxide semiconductor
ECT: electron cryotomography
FRAP: fluorescence recovery after photo-bleaching
IPTG: isopropyl β-D-1-thiogalactopyranoside
PALM: photoactivated localization microscopy
SEM: standard error of the mean
SIM: structured illumination microscopy
STED: stimulated emission depletion
