# Supplementary figures and images for: 3D-SIM Super Resolution Microscopy Reveals a Bead-Like Arrangement for FtsZ and the Division Machinery: Implications for Triggering Cytokinesis
Source: PLoS Biol. 2012 Sep 11;10(9):e1001389. doi: 10.1371/journal.pbio.1001389 (PMC3439403; doi:10.1371/journal.pbio.1001389)

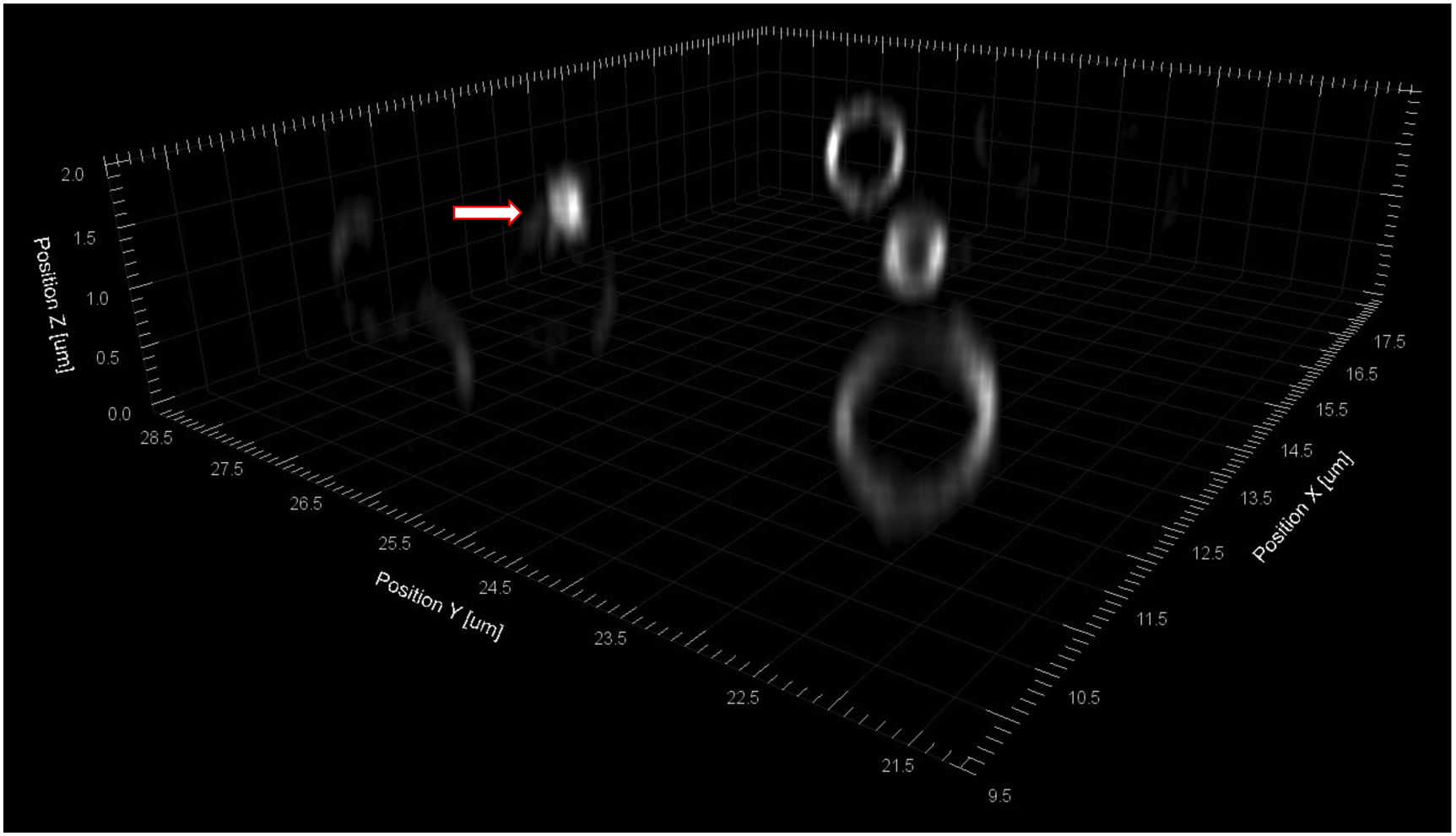

Supplement: Figure S1 — Non-constricting and constricting Z ring appearance in live cells of SU570 using 3D-SIM (OMX V3). Visualizing the heterogeneous distribution of FtsZ-GFP is easier in Z rings with a diameter between ∼0.3 and 0.9 µm. Constricting Z rings (white arrow) with a diameter less than 0.3 µm are harder to visualize any heterogeneous distribution of FtsZ-GFP. (TIF) [file pbio.1001389.s001.tif]

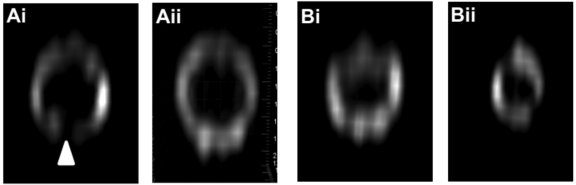

Supplement: Figure S2 — Immunofluorescence labeling of wild-type B. subtilis cells (SU5) reveals a similar heterogeneous distribution of FtsZ inside the Z ring. (Ai–Aii) 1∶100 dilution of anti-FtsZ. Z ring diameter, 0.9 µm (Bi–Bii) 1∶10,000 dilution of anti-FtsZ. Z ring diameter, 0.7 µm and 0.5 µm, respectively. Cells were grown at 30°C in PAB and imaged using 3D-SIM (OMX V3). (TIF) [file pbio.1001389.s002.tif]

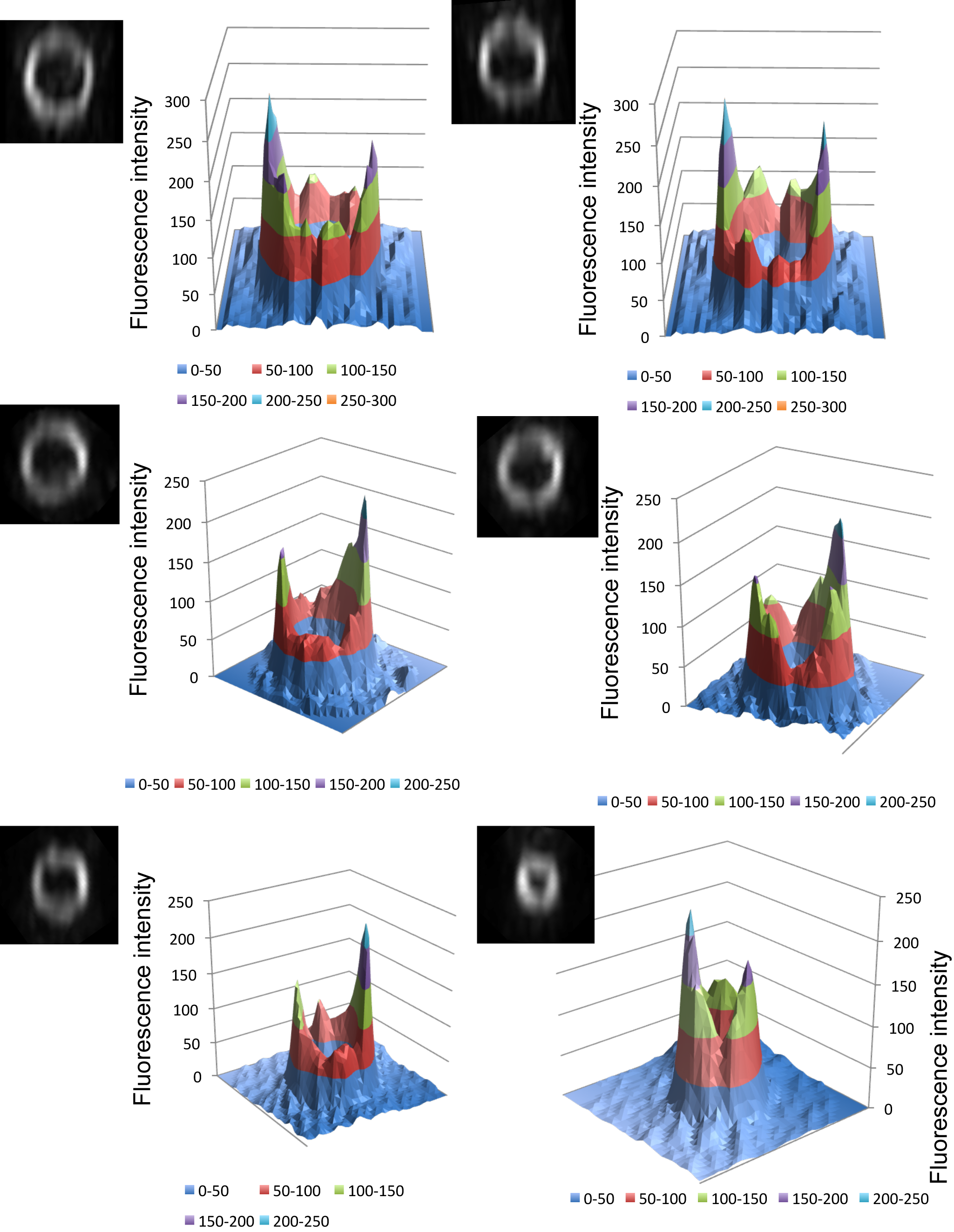

Supplement: Figure S3 — Additional examples of 3D intensity plots of B. subtilis Z rings. See Figure 4. SU570 cells were imaged using 3D-SIM (OMX V3). (TIF) [file pbio.1001389.s003.tif]
